# Common Variable Immunodeficiency and Autoimmune Diseases: A Retrospective Study of 95 Adult Patients in a Single Tertiary Care Center

**DOI:** 10.3389/fimmu.2021.652487

**Published:** 2021-07-05

**Authors:** Ilaria Mormile, Alessandra Punziano, Carlo Alberto Riolo, Francescopaolo Granata, Michela Williams, Amato de Paulis, Giuseppe Spadaro, Francesca Wanda Rossi

**Affiliations:** ^1^ Department of Translational Medical Sciences, University of Naples Federico II, Naples, Italy; ^2^ Post-Graduate Program in Clinical Immunology and Allergy, University of Naples Federico II, Naples, Italy; ^3^ Center for Basic and Clinical Immunology Research (CISI), WAO Center of Excellence, University of Naples Federico II, Naples, Italy

**Keywords:** common variable immunodeficiency, autoimmunity, arthritis, cytopenia, psoriasis

## Abstract

Common variable immunodeficiency (CVID) is the most common clinically significant primary immunodeficiency in adulthood, which presents a broad spectrum of clinical manifestations, often including non-infectious complications in addition to heightened susceptibility to infections. These protean manifestations may significantly complicate the differential diagnosis resulting in diagnostic delay and under-treatment with increased mortality and morbidity. Autoimmunity occurs in up to 30% of CVID patients, and it is an emerging cause of morbidity and mortality in this type of patients. 95 patients (42 males and 53 females) diagnosed with CVID, basing on ESID diagnostic criteria, were enrolled in this retrospective cohort study. Clinical phenotypes were established according to Chapel 2012: i) no other disease-related complications, ii) cytopenias (thrombocytopenia/autoimmune hemolytic anemia/neutropenia), iii) polyclonal lymphoproliferation (granuloma/lymphoid interstitial pneumonitis/persistent unexplained lymphadenopathy), and iv) unexplained persistent enteropathy. Clinical items in the analysis were age, gender, and clinical features. Laboratory data included immunoglobulin (Ig)G, IgM and IgA levels at diagnosis, flow-cytometric analysis of peripheral lymphocytes (CD3+, CD3+CD4+, CD3+CD8+, CD19+, CD4+CD25highCD127low, CD19hiCD21loCD38lo, and follicular T helper cell counts). Comparisons of continuous variables between groups were performed with unpaired t-test, when applicable. 39 patients (41%) showed autoimmune complications. Among them, there were 21 females (53.8%) and 18 males (46.2%). The most prevalent autoimmune manifestations were cytopenias (17.8%), followed by arthritis (11.5%), psoriasis (9.4%), and vitiligo (6.3%). The most common cytopenia was immune thrombocytopenia, reported in 10 out of 95 patients (10.5%), followed by autoimmune hemolytic anemia (n=3, 3.1%) and autoimmune neutropenia (n=3, 3.1%). Other autoimmune complications included thyroiditis, coeliac disease, erythema nodosum, Raynaud’s phenomenon, alopecia, recurring oral ulcers, autoimmune gastritis, and primary biliary cholangitis. There were no statistically significant differences comparing immunoglobulin levels between CVID patients with or without autoimmune manifestations. There was no statistical difference in CD3+, CD8+, CD4+CD25highCD127low T, CD19, CD19hiCD21loCD38lo, and follicular T helper cell counts in CVID patients with or without autoimmune disorders. In conclusion, autoimmune manifestations often affect patients with CVID. Early recognition and tailored treatment of these conditions are pivotal to ensure a better quality of life and the reduction of CVID associated complications.

## Introduction

Common variable immunodeficiency (CVID) is the most common clinically significant primary immunodeficiency (PID) in adulthood (1). The diagnosis of CVID is established after the 4^th^ year of age (but symptoms may be present earlier) and based on the following criteria (2): marked decrease of immunoglobulin (Ig)G, IgA and/or IgM as compared with age-related standard, impaired or absent antibody production, exclusion of secondary causes of hypogammaglobulinemia, and no evidence of profound T-cell deficiency. The European Society for Immunodeficiencies (ESID) diagnostic criteria (ESID Registry-Working definitions for clinical diagnosis of PID) are available from (http://esid.org/Working-Parties/Registry/Diagnosis-criteria).

Over 90% of CVID patients suffer from severe, recurrent, and sometimes chronic bacterial infections mainly of the respiratory and gastrointestinal tracts (3); other common clinical presentations are granulomatous diseases and unexplained polyclonal lymphoproliferation (3).

In recent years, the quality of life and prognosis of patients with CVID have improved thanks to advances in the management and prophylaxis of infections with antibacterial agents and immunoglobulin replacement therapy (IgRT). Simultaneously, there has been an increased awareness of autoimmunity as an emerging cause of morbidity and mortality (4).

Autoimmunity occurs in up to 30% of CVID patients (5–7), and it is frequently the presenting manifestation at the onset of immunodeficiency (1). In addition, in a study by Quinti et al. (8) autoimmunity was found in 17.4% of 224 patients with CVID, and in 2.3% of these patients, it was the only clinical manifestation at the diagnosis of CVID. Cytopenias are the most common autoimmune conditions described in CVID (6, 9, 10). Immune thrombocytopenia (ITP) is the most frequent autoimmune cytopenia, with a 7% to 14% prevalence, while the proportion of CVID patients with autoimmune hemolytic anemia (AIHA) and autoimmune neutropenia is 4-7% and 1%, respectively (6). Rheumatologic diseases observed in CVID patients are rheumatoid arthritis (2.6-3.6%), Sjögren’s syndrome (<1-4.2%), and systemic lupus erythematosus (<1%) (3). Other autoimmune conditions related to CVID are vitiligo (<1-3.9%), autoimmune thyroiditis (<1-3.9%), diabetes mellitus (<1-3.9%), multiple sclerosis (<1-3.9%), alopecia (1.1-1.6%), and pernicious anemia (<1-1.2%) (3).

Our study aims to characterize the clinical phenotype and immunological findings of CVID patients with associated autoimmune complications. CVID may indeed present a broad clinical spectrum, therefore preventing diagnostic delay may sometimes be very challenging (11), especially due to the lack of physicians’ awareness about some atypical manifestations of this disease.

## Materials and Methods

### Patients

A total of 95 adult patients (42 males and 53 females) diagnosed with CVID at the Division of Allergy and Clinical Immunology of the University of Naples Federico II, Naples, Italy, were enrolled in this retrospective cohort study. Clinical and laboratory data were retrospectively collected until December 2020. We included patients with a CVID diagnosis based on the ESID diagnostic criteria (available at http://esid.org/Working-Parties/Registry/Diagnosis-criteria), available data on sex, date of birth, age of onset, CVID diagnosis age, serum levels of immunoglobulins (IgG, IgA, and IgM) at diagnosis, and signature of the written informed consent. Patients under 18 years of age were excluded. Secondary causes of hypogammaglobulinemia (e.g., drugs, malignancies) were ruled out. We also excluded four CVID patients treated with rituximab for granulomatous and lymphocytic interstitial lung disease (GLILD). Patients diagnosed with CVID developing malignancies in clinical remission at the time of enrolment were included.

Less than 20% of cases of CVID patients have a known underlying genetic cause (12). In our cohort six patients underwent genetic tests, in four patients no genetic defects were found and two patients exhibit TACI mutations.

### Clinical Phenotype

Clinical phenotypes were established according to Chapel 2012 (13, 14): i) no other disease-related complications, ii) cytopenias (thrombocytopenia/autoimmune hemolytic anemia/neutropenia), iii) polyclonal lymphoproliferation (granuloma/lymphoid interstitial pneumonitis/persistent unexplained lymphadenopathy), and iv) unexplained persistent enteropathy.

Splenomegaly and malignancies were excluded as phenotyping criteria according to Chapel 2012 (13, 14) and described separately. Indeed, splenic enlargement may be due to various causes rather than a relationship with the underlying disease (13), similarly, immunodeficiency could develop as a secondary clinical event of malignancies (i.e., lymphoid malignancies) (14).

In addition, we described other significant clinical features in our cohort, such as psoriasis, vitiligo, thyroiditis, coeliac disease, erythema nodosum, Raynaud’s phenomenon, alopecia, recurring oral ulcers, autoimmune gastritis, and primary biliary cholangitis.

### Laboratory Studies

Laboratory data included IgG, IgM, and IgA serum levels at the diagnosis and flow-cytometric analysis (BD FACS Canto II, Erembodegem, Belgium) of peripheral lymphocytes [CD3+, CD3+CD4+, CD3+CD8+, CD19+, CD4+CD25highCD127low T regulatory (Treg), CD19hiCD21loCD38lo B cells count, follicular helper T cells].

**Figure 1:**
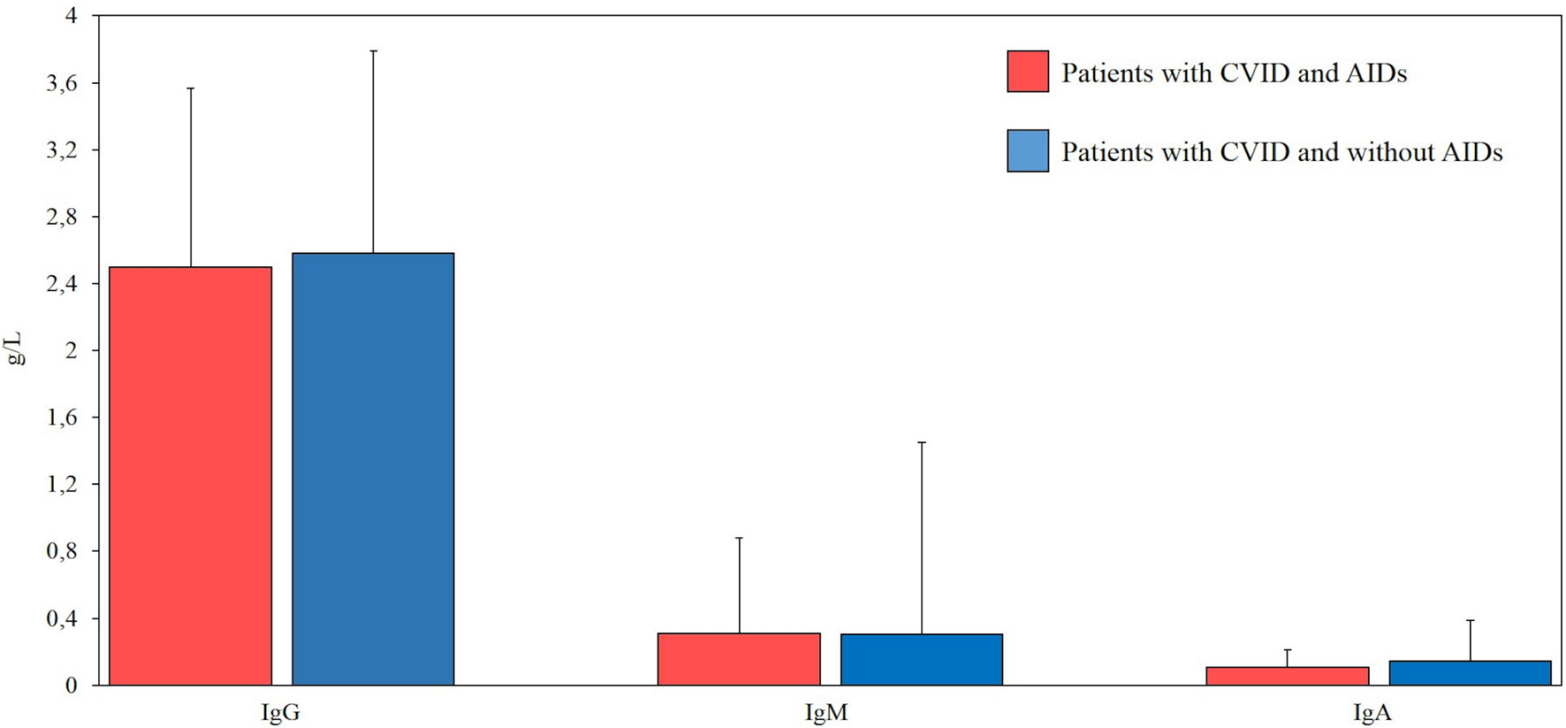
Comparison between serum levels of immunoglobulins (g/L) at diagnosis in our cohort of patients with common variable immunodeficiency (CVID) and autoimmune diseases (AIDs) (red bar, n=39) and CVID without AIDs (blue bar, n=56). Normal ranges (g/l) for IgG (7.37-16.07), IgM (0.40-2.30), IgA (0.70-4).

All patients had available data on sex, date of birth, age of onset, and CVID diagnosis age. Furthermore, serum levels of immunoglobulins (IgG, IgA, and IgM) at diagnosis, before IgRT treatment, were available for all 95 CVID patients (**Figure 1**).

**Figure 2:**
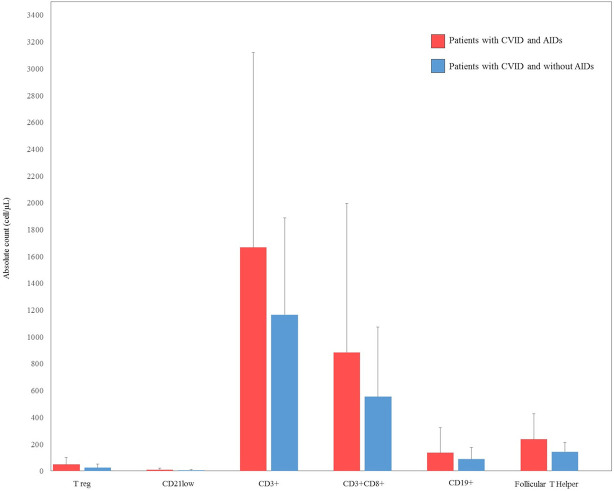
Comparison between lymphocyte count subsets at diagnosis in our cohort of patients with common variable immunodeficiency (CVID) and autoimmune diseases (AIDs) (red bar) and CVID without AIDs (blue bar). CD4+CD25highCD127low T regulatory (Treg), CD19hiCD21loCD38lo B cells (CD21low).

There were some missing data in the lymphocyte subsets counts. CD3+ were available for 26 (66%) patients with autoimmune complications, and for 25 (44%) CVID patients without autoimmune complications. CD8+ T cells and CD19+ cells were available for 26 (66%) patients with autoimmune complications, and for 23 (41%) CVID patients without autoimmune complications. CD4+CD25highCD127low Treg were available for 20 (51%) patients with autoimmune complications, and for 22 (39%) CVID patients without autoimmune complications. CD19hiCD21loCD38lo B cells were available for 19 (48%) patients with autoimmune complications and for 18 (32%) CVID patients without autoimmune complications. Follicular T helper cells were available for 15 (28%) patients with autoimmune complications and for 13 (23%) CVID patients without autoimmune complications. Cell counts are displayed as the absolute number of cell/microliter (cell/µL) (**Figure 2**).

Due to the hypogammaglobulinemic condition and defect in specific antibodies response in CVID patients, autoantibodies were considered not significant for establishing the diagnosis of autoimmune diseases (15).

### Data Analysis

Data were analyzed using the software GraphPad Prism 8. Values were presented as frequency (number and percentage) and mean ± standard error of the mean (SEM). Comparisons of continuous variables between groups were performed with unpaired t-test, when applicable. A significance level of *p ≤* 0.05 was assumed for all statistical evaluations.

## Results

### Demographic Data

95 CVID patients, 42 males (44.2%) and 53 females (55.8%), were enrolled in this study. The cohort was White-Caucasian. The average age at diagnosis was 52 years (24–83). The patients were followed for an average time of 11.56 years (1–35). 10 patients died during follow-up.

### Clinical Evaluation

**Figure 3:**
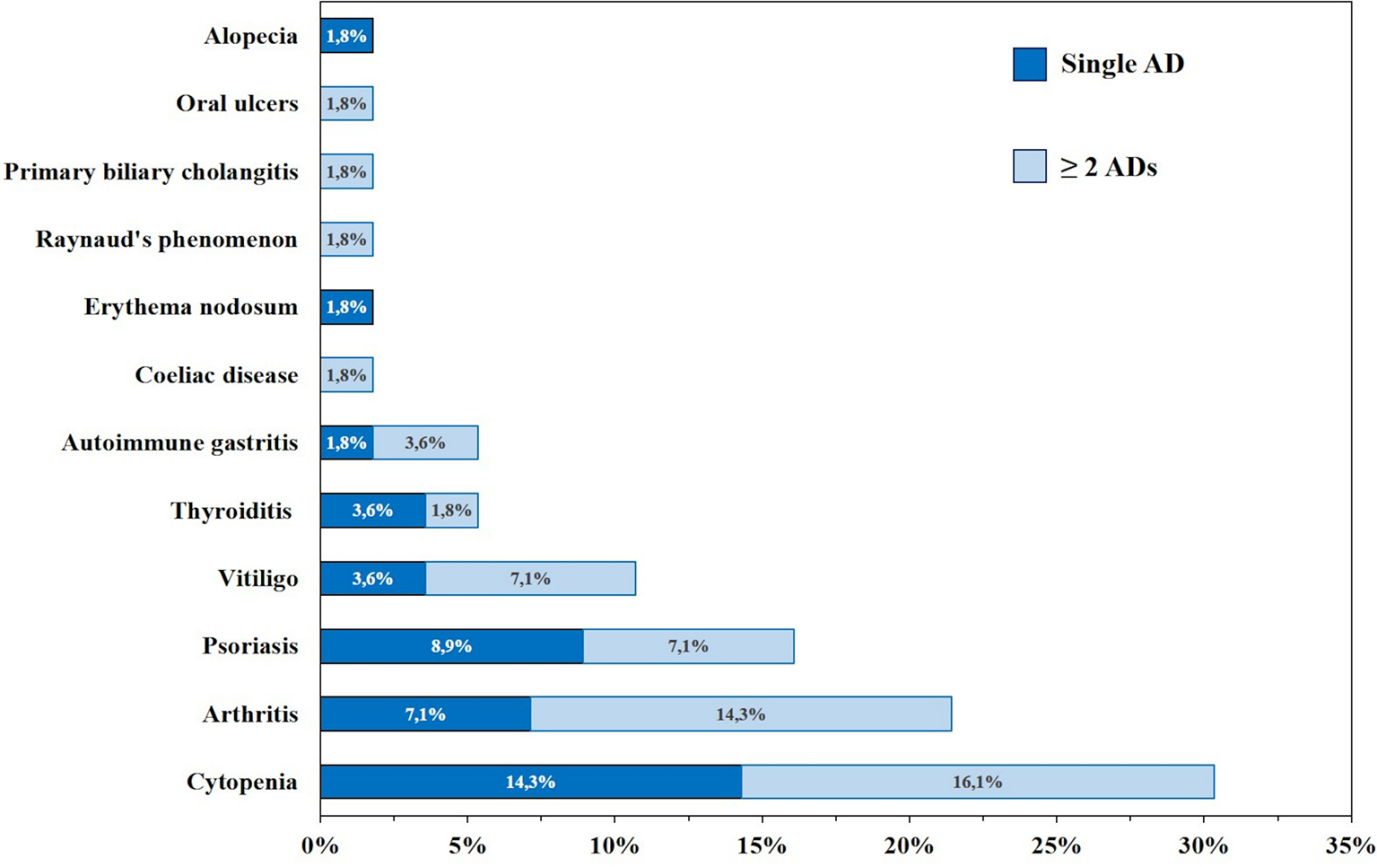
Frequency (%) of autoimmune manifestations presenting alone (dark blue) or in association (light blue) in patients with common variable immunodeficiency (CVID) (n=95).

Thirty-nine patients (41%) showed autoimmune complications. Among them were 21 females (53.8%) and 18 males (46.2%). The frequency of the most common autoimmune manifestations in our cohort is summarized in **Table 1** and **Figure 3**. Other autoimmune complications included coeliac disease, erythema nodosum, Raynaud’s phenomenon, alopecia, recurring oral ulcers, autoimmune gastritis, and primary biliary cholangitis (**Table 1** and **Figure 3**).

**Table 1:** Distribution of autoimmune disease in 95 CVID patients.

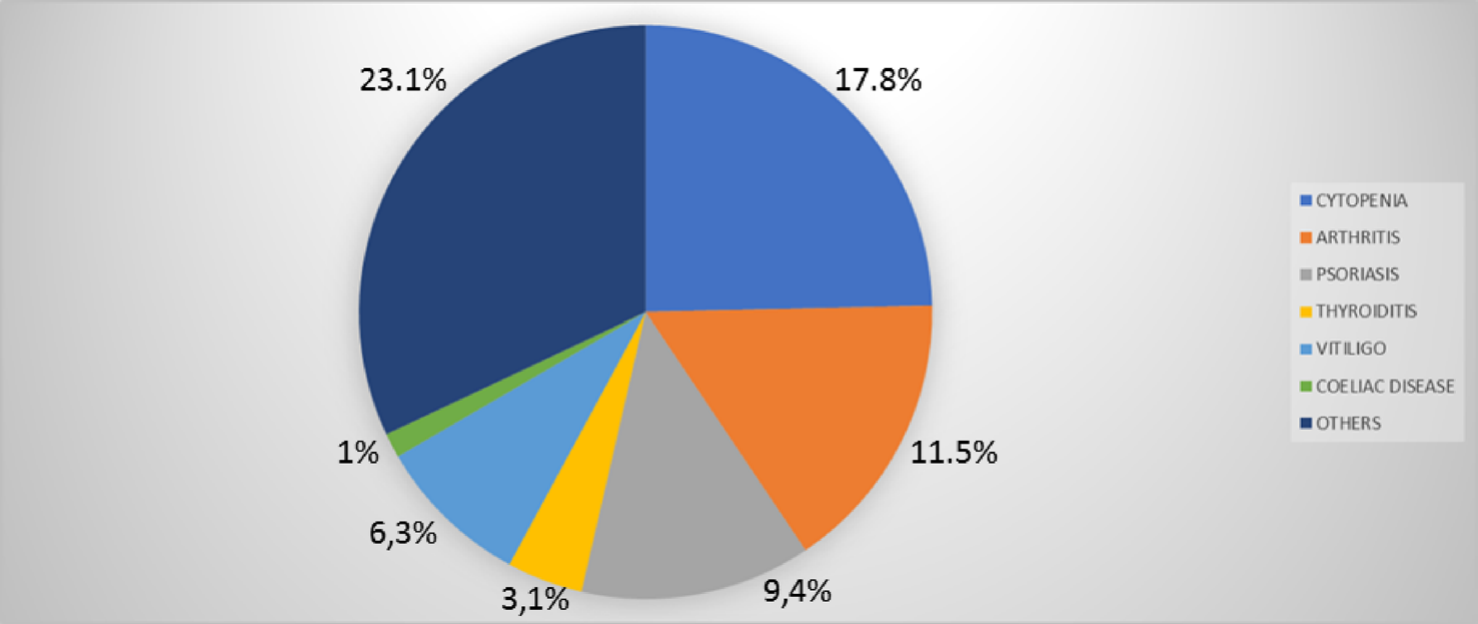		
Autoimmune manifestations	Patient (Number)	Frequency (%)
Cytopenia	17	17.8%
Arthritis	11	11.5%
Psoriasis	9	9.4%
Vitiligo	6	6.3%
Thyroiditis	3	3.1%
Coeliac disease	1	1%
Others	22	23.1%
**Total**	**39 out of 95**	**41%**

**Figure 4:**
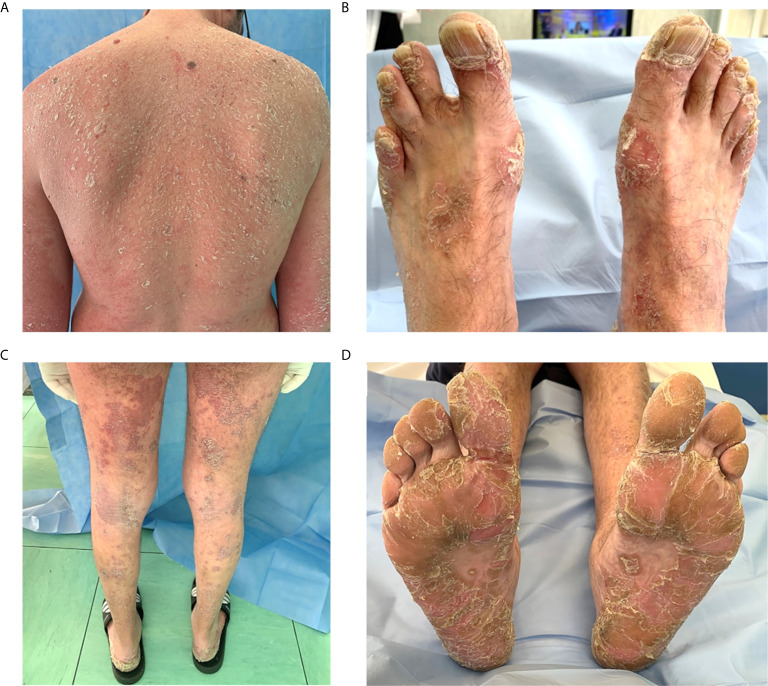
Severe psoriasis in a patient with common variable immunodeficiency. **(A–D)** Erythrodermic psoriasis diffuse to the entire body with a red and peeling rash. **(B)** Nail psoriasis is evident at toenails, causing pitting, discoloration and onycholysis. **(C)** Symmetrical erythematous plaques with sharp edges and covered with pearlescent scales, located on the posterior surface of the legs. **(D)** Palmoplantar psoriasis involving symmetrically palms of the hands and soles of the feet; squamae are the predominant lesions.

Almost all patients (7 out of 9; 7%) affected by psoriasis presented a mild form. Two patients showed a diffuse cutaneous involvement, with nail psoriasis. One patient showed a severe form of erythrodermic psoriasis involving the entire body surface with palmoplantar psoriasis and onycholysis (**Figure 4**).

Among 11 patients presenting with arthritis (11.5%), nine patients showed a rheumatoid-like phenotype, and two patients were diagnosed with psoriatic arthritis, based on clinical, laboratory, and radiological findings (16, 17). We included in this study only patients with aseptic inflammatory arthritis (16, 17).

We also compared prevalence of autoimmune manifestations in our cohort and the general prevalence on Italian population; results are reported in **Table 2**. Clinical phenotypes were established according to Chapel 2012 classification (14).

**Table 2:** Prevalence of autoimmune manifestations in our CVID cohort (n=95) vs Italian general population.

Autoimmune manifestations	Prevalence (%) in CVID patients (n=95)	Prevalence (%) in Italian general population (data reference)
**Cytopenia**	17.8	6.8 (18)
**Rheumatoid arthritis**	9.4	0.5 (19)
**Psoriatic arthritis**	2.1	4.7-47.1 (20)
**Psoriasis**	9.4	1.8-3.1 (20)
**Vitiligo**	6.3	0.17 (21)
**Thyroiditis**	3.1	3 (22)
**Coeliac disease**	1	0.4 (23)
**Autoimmune gastritis**	3.1	2-5^a^ (24)
**Alopecia**	1	1-2 (25)
**Primary biliary cholangitis**	1	0.03 (26)
**Raynaud’s phenomenon**	1	3.4^b^ (27)

a Prevalence in Western countries.

b Prevalence of Raynaud’s phenomenon in women.

**Figure 5:**
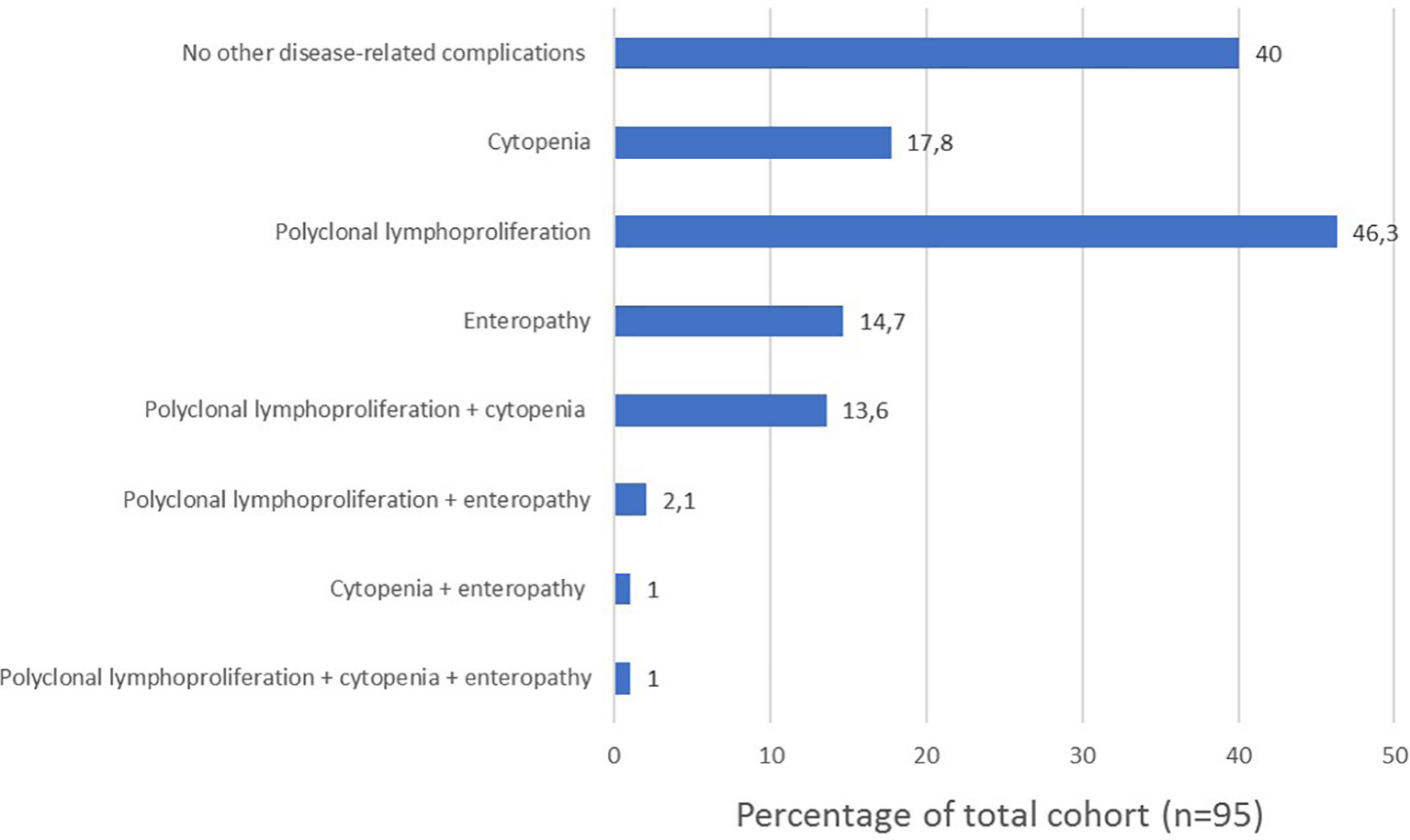
Frequency of clinical phenotypes and their overlap in our cohort (n=95).

Phenotypes are not mutually exclusive as shown in **Figure 5**. Among patients with autoimmunity, 13 patients (13.6%) presented with the no other disease-related complications phenotype; 17 (17.8%) presented with the cytopenia phenotype; 22 (22.9%) presented with the polyclonal lymphoproliferation phenotype; 7 (7.3%) presented with the enteropathy phenotype.

Among patients with arthritis seven had no other disease-related complications (63.6%), three patients presented cytopenia, three patients polyclonal lymphoproliferation, and one patient had enteropathy. Clinical manifestations associated with autoimmune cytopenias were polyclonal lymphoproliferation (n=14, 82.3%), enteropathy (n=5, 29.4%), arthritis (n=3, 17.6%), thyroiditis (n=1, 5.8%), coeliac disease (n=1, 5.8%), autoimmune gastritis (n=1, 5.8%), vitiligo (n=1, 5.8%), and primary biliary cholangitis (n=1, 5.8%). 14 out of 95 (14.7%) patients presented malignancy during the follow-up. The most common malignancy in our cohort was non-Hodgkin’s lymphoma (n=4, 4.2%), followed by skin cancer (n=3, 3.15%; two patients with basal cell carcinoma and one patient with squamous cell carcinoma). Other malignancies found in our cohort were Hodgkin’s lymphoma, breast, gastric, thyroid, gallbladder, and nasopharyngeal cancers.

The overall prevalence of splenomegaly in our cohort was 47.3% (n=45) and it was found in 21 out of 39 patients (53.8%) with CVID and autoimmunity; most of the patients with splenomegaly had autoimmune cytopenias (15 out of 21; 71.4%). On the other hand, almost all patients with autoimmune cytopenia had splenomegaly (15 out of 17; 88%), in line with the observation that splenomegaly is more common in patients with cytopenias, although this association is not clearly understood (28). Granulomatous and lymphocytic interstitial lung disease (GLILD) was observed in 12 patients (12.6%).

### Treatment

All patients were treated with intravenous (76 patients) or subcutaneous (19 patients) IgRT (9, 29, 30). To manage rheumatologic complications four patients were treated with medium-low doses of glucocorticoids (≤10 mg/day), two patients with hydroxychloroquine (200-400 mg/day), two patients with sulfasalazine (2 g/day) and one patient, who failed therapy with hydroxychloroquine and cyclosporine (140 mg/day), was switched to methotrexate (7.5 mg/week) which led to symptom improvement.

Four patients with autoimmune thrombocytopenia started therapy with glucocorticoids up to at 1 mg/kg prednisone-equivalent. In addition, one patient who presented steroid-dependency underwent splenectomy and high-dose intravenous immunoglobulin (IVIG) (1–2 g/kg in 5 days) was administrated, with good clinical outcome. Clinical features of patients with CVID and autoimmunity are displayed in **Table 3**.

**Table 3:** Clinical features in our cohort of patients with CVID and autoimmune manifestations.

Patient	Age (Years)	Sex (M/F)	Clinical Phenotype [2]	Autoimmune Disease
1	30	F	C, PL, E	ITP
2	46	M	C, PL	ITP, autoimmune neutropenia
3	26	F	I	Autoimmune gastritis
4	56	M	PL, E	Thyroiditis
5	37	M	C, PL, E	ITP, psoriasis
6	54	F	C, PL	ITP
7	64	M	I	Arthritis, thyroiditis
8	59	M	PL	Thyroiditis
9	58	M	C	AIHA
10	50	M	C, E	ITP, arthritis, coeliac disease
11	74	M	PL	Autoimmune gastritis, vitiligo
12	75	M	E	Vitiligo
13	36	F	C, PL	Autoimmune gastritis, AIHA
14	43	M	I	Alopecia
15	57	M	I	Arthritis
16	71	F	C, PL	Arthritis, ITP, psoriasis
17	61	M	C, PL	Autoimmune neutropenia
18	50	M	I	Arthritis
19	27	M	C, PL	Arthritis, other cytopenias
20	65	F	I	Arthritis, psoriasis
21	58	M	I	Arthritis
22	47	F	C, E	Arthritis, ITP
23	69	F	C, PL	ITP, AIHA
24	67	F	C, PL	ITP, AIHA, Autoimmune neutropenia
25	53	F	I	Arthritis
26	58	F	I	Vitiligo
27	64	F	I	Arthritis, Raynaud’s phenomenon
28	52	F	PL	Vitiligo, recurring oral ulcers, psoriasis
29	76	F	C, PL	Vitiligo, other cytopenias
30	81	F	PL	Vitiligo, arthritis
31	45	F	I	Erythema nodosum
32	58	F	C, PL	ITP, primary biliary cholangitis
33	24	M	C, PL, E	ITP
34	30	M	C, PL	ITP
35	39	F	I	Psoriasis
36	30	F	PL	Psoriasis
37	32	F	I	Psoriasis
38	53	F	PL	Psoriasis
39	38	M	PL	Psoriasis

I, no other disease-related complications; C, cytopenias; PL, polyclonal lymphoproliferation; E, unexplained persistent enteropathy; ITP, immune thrombocytopenia; AIHA, Autoimmune hemolytic anemia.

### Immunological Investigations

Serum immunoglobulin concentrations and CD3+, CD8+, CD4+CD25highCD127low, CD19, CD19hiCD21loCD38lo and follicular T helper cell counts were compared between CVID patients with and without autoimmune disorders. No statistically significant differences were observed in the immunoglobulin levels between the two groups of patients. There was no statistical difference in CD3+, CD8+, CD4+CD25highCD127low T reg, CD19, CD19hiCD21loCD38lo, and follicular T helper cells in CVID patients with or without autoimmune disorders. Laboratory findings of patients with CVID and autoimmune manifestations are summarized in **Figures 1** and **2**.

## Discussion

The coexistence of immunodeficiency with autoimmunity may appear oxymoronic, in particular it is unclear how autoantibodies can be produced in a state of antibody deficiency (1). On the one hand, the production of antibodies of patients with CVID is severely impaired or completely absent, and, on the other hand, patients with CVID may show features consistent with an overactive immune system (31, 32). To understand this relationship, it must be considered that the immune system is in constant balance with stimulatory and inhibitory factors that affect common immune cells (31). In addition, it has been shown that the development of autoimmunity in these patients is the consequence of a lower efficacy of the self-tolerance mechanisms, linked in turn to alterations of the immune-regulatory mechanisms (32). Based on this suggested pathogenetical mechanism, several research groups have tried to identify some common immunophenotipical features in patients with CVID associated with autoimmune manifestations. The composition of the B cell compartment had been previously used to classify CVID patients to predict clinical phenotype and complications (7, 33–36). An expansion in CD21low B cells has been found in a subgroup of CVID patients with autoimmunity by several research groups (7, 35, 37), as found in other autoimmune diseases (38, 39). Rakhmanov et al. (40) demonstrated that in CVID, CD21low B cells are a polyclonal, pre-activated, partially autoreactive, functionally attenuated B cell population with preferential enrichment in peripheral tissues, like the bronchoalveolar space of CVID or the synovium of rheumatoid arthritis patients. In addition, CD21low B cells produce significantly higher amounts of IgM as compared to näive B cells upon stimulation with CD40L, IL-2 and IL-10, and higher IgM levels have been associated with the development of autoimmunity in CVID patients (5, 7, 28, 41). Several studies have demonstrated that T cells play a significant role in CVID pathogenesis, overtaking the old-fashioned concept that CVID is exclusively due to B cell defects (4), and their abnormalities have been analyzed in patients with associated autoimmune diseases. CVID patients with autoimmunity generally present with lower total T cells than those without autoimmunity (4, 42). Among T cell subtypes, autoimmunity has particularly been correlated with a reduction in Treg cells and an increase in T follicular helper CD4+ cells (43, 44). Indeed, patients with CVID and autoimmune cytopenia show irregularly shaped hyperplastic germinal centers with an increased number of circulating T follicular helper cells, which likely play a role in developing autoreactive B cells (31, 45). In our cohort, we compared Ig levels or lymphocyte subsets between CVID patients with or without autoimmune disorders or arthritis, but we did not find any statistical difference between these groups. However, there was a large amount of missing data for lymphocyte subsets counts, and this could represent an important limitation of our analysis and should be considered in the interpretation of our data. Despite that, it should be emphasized that other previously reported analysis of the immunophenotypic parameters in patients with CVID-associated autoimmune diseases leaded to controversial results. For instance, Gutierrez et al. (46) did not demonstrate significant differences concerning IgA and IgM levels, CD19+ B-cell counts and CD4/CD8 ratio between groups of patients with CVID associated with rheumatologic manifestations and patients with CVID without autoimmune complications.

The prevalence of autoimmune diseases in our study population was 41% (8). Fischer et al. (4) demonstrated that autoimmune and inflammatory diseases are much more frequent in patients with PIDs than in the general population. Data from the ESID registry analyzing a cohort of 2,700 CVID patients reported that especially autoimmune cytopenia was 700 times more prevalent in CVID patients than the general population (47). In addition, it is frequently the presenting manifestation at the onset of immunodeficiency (1) preceding CVID diagnosis by several years in up to 60% of patients (48). ITP is the most common autoimmune cytopenia in CVID with a 7% to 14% prevalence, while AIHA and autoimmune neutropenia respectively occur in 7% and 1% of CVID patients (6). Cytopenia has been reported as the only autoimmune condition associated with a decreased survival, therefore, phenotyping CVID patients, it is considered separately from autoimmunity in general (14, 42).

Our whole cohort was classified according to Chapel 2012 clinical phenotypes (14). The phenotype overlap we described align with those observed by Chapel et al. (13, 14). The authors analyzed data from 334 patients with CVID from 7 European centers to distinguish clinical phenotypes and observed that 83% of the patients had only one clinical phenotype.

However, other research groups have investigated CVID clinical features association (**Table 4**) and reported that phenotype overlap might be more common. The most contrasting results were obtained by Selenius et al. (49) analyzing a Finnish cohort of 132 patients (106 diagnosed with “probable” and 26 with “possible” CVID) and describing the concomitance of multiple phenotypes in 73% of patients with probable CVID (49). The most common phenotype overlap we observed was the association between polyclonal lymphoproliferation and cytopenia phenotype. Feuille et al. (32) analyzed data from the United States Immunodeficiency Network (USIDNET) Registry to characterize phenotypes associated with autoimmune cytopenias in 990 patients with CVID. The authors found that CVID patients with autoimmune cytopenia were more likely to have other CVID-associated non-infectious complications (OR= 2.9; 95%-CI: 1.9–4.6, P<0.001), including lymphoproliferation, lymphomas, granulomatous disease, hepatic disease, interstitial lung diseases, enteropathy, and organ-specific autoimmunity. These results align with those observed in our study (**Tables 1** and **3**).

**Table 4:** Prevalence of CVID clinical phenotypes and their overlap in geographically different cohorts.

Reference	Country	Total CVID patients (N)	Clinical Phenotype	Prevalence (%)	Phenotypes overlap prevalence (%)
(13)	Czech Republic	41	A	29%	17%
			PL	54%	
			E	6%	
			HM	0%	
			I	34%	
	Germany	68	A	38%	
			PL	31%	
			E	10%	
			HM	6%	
			I	40%	
	Sweden	129	A	27%	
			PL	12%	
			E	3%	
			HM	0.8%	
			I	61%	
	United Kingdom	96	A	48%	
			PL	39%	
			E	13%	
			HM	5%	
			I	37%	
(9)	Europe	2212	A	29%	NR
			E	9%	
			HM	3%	
			SM	5%	
			G	9%	
(49)	Finland	106 “Probable CVID” (50)	A	51%	73%
			PL	70%	
			E	20%	
			M	14%	
			I	15%	
		26 “Possible CVID” (50)	A	35%	NR
			PL	35%	
			E	9%	
			M	19%	
			I	36%	
(32)	USA	990	C	10.2%	69.4%^*^
			PL	19.8%	
			E	5.6%	
			HM	4.3%	
This study	Italy	95	C	17.8%	18%
			PL	46.3%	
			E	14.7%	
			I	40%	

A, autoimmunity; C, cytopenias; E, enteropathy; G, granuloma; HM, hematologic malignancy; I, no other disease-related complications; M, malignancies; PL, polyclonal lymphoproliferation; SM, solid tumors; NR, not reported.

^*^Association between autoimmune cytopenia and non-infectious CVID-associated conditions.

Dermatological involvement including alopecia totalis, lichen planus, and vitiligo has occasionally been reported in CVID (31). Psoriasis typically affects approximately 2% to 4% of the general population (51) and it has been considered at length an uncommon manifestation in CVID, seen in less than 1% of patients (52). However, data regarding frequency and features of psoriasis in CVID patients were previously limited to few case reports. Gualdi et al. (53) reported for the first time that psoriasis prevalence was much higher in the CVID patients (9/47 patients; 19.14%) than the general population. The cause of this higher prevalence is not well-known but contributing factors may include T-cell imbalance or other immune-related dysregulation mechanisms (53). A recent one-year observational prospective study carried out by our research group (54) performed a complete dermatological examination on 58 patients with CVID describing psoriasis in 13 out of 58 patients (22%). Interestingly almost all patients (12 of 13; 92%) presented a mild form of psoriasis, possibly due to the immunomodulatory effects of IgRT on dendritic cells, regulatory T cells, B cells, and NK cells functions, which are involved in the pathogenesis of the dermatosis (54, 55). In addition, marked clinical improvement in psoriasis has been observed after the initiation of IgRT, which has a significant role in the management of psoriasis in CVID patients.

Rheumatologic disorders are also frequently described in CVID patients (46) and chronic inflammatory arthritis occurs in approximately 3% of CVID patients (6, 10, 41, 46). The most frequent joint manifestation in CVID is aseptic, possibly immune-mediated arthritis (1-10%), characterized by a symmetrical involvement of limited or several joints, especially knees, ankles, and hands (56). However, septic arthritis should always be excluded, as patients with CVID can often present arthritis due to extracellular bacteria, encapsulated bacteria (*Streptococcus pneumoniae*, *Haemophilus influenzae*, and *Staphylococcus aureus*), or even atypical bacteria *(Mycoplasma hominis*, *Mycoplasma pneumoniae*, *Mycoplasma salivarium*, and *Ureaplasma urealyticum*), as well as enteroviruses (16, 17). Differential diagnosis should also include amyloidosis; indeed, in CVID patients with recurrent infections, amyloidosis is a potential cause of joint inflammation (31).

**Figure 6:**
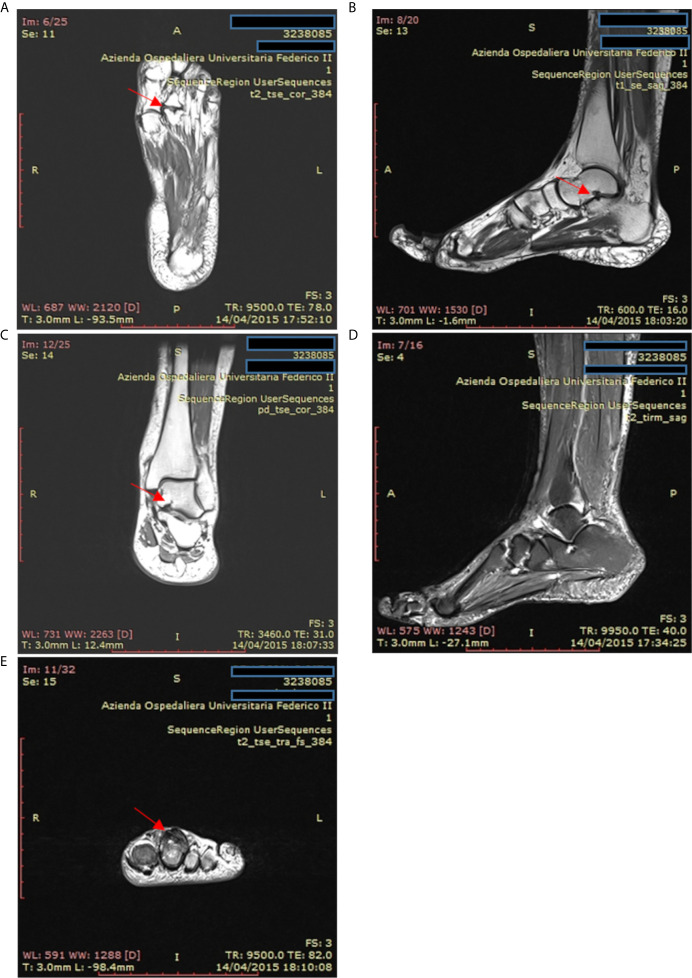
Magnetic resonance imaging of the ankle and foot in a patient with psoriatic arthritis and common variable immunodeficiency. **(A)** T2-weighted coronal magnetic resonance images of the left foot in a patient with psoriatic arthritis. Erosions are present at the second metatarsal-phalangeal joint (arrow). **(B, C)** T1-weighted sagittal axis and coronal magnetic resonance images showing cystic erosion (7 mm) of the floor of the sinus tarsi (arrow). **(D)** T2-weighted sagittal magnetic resonance images of foot and ankle showing joint effusion in the tibiotarsic compartment and enthesitis at the Achilles tendon insertion. **(E)** T2-weighted axial magnetic resonance images of the fingers from a patient with psoriatic arthritis exhibiting flexor and extensor tenosynovitis at the second finger (arrow).

Accordingly, the most common rheumatologic condition in our CVID patients was inflammatory arthritis, but we also reported erythema nodosum and Raynaud’s phenomenon in 2 out of 39 patients. Conventional radiography and Magnetic Resonance Imaging are useful diagnostic tools to assess the diagnosis and to identify erosive and non-erosive arthritis, as described in one patient included in our study (**Figure 6**). On the contrary, antinuclear antibodies and rheumatoid factor are typically absent in CVID (31). Synovial biopsies show synovial hyperplasia and capillary proliferation; the classic synovial infiltrates of B lymphocytes and polymorphonuclear leukocytes are usually absent, while the T infiltrates are mainly composed of CD8+ T cells (57).

The treatment of rheumatological disease in CVID is almost the same as that of primary autoimmune disease. Glucocorticoids represent the first line of treatment for all the complications of CVID associated with immune dysregulation. Other treatment strategies, administrated in combination with IgRT (9, 29, 30), include Disease Modifying Antirheumatic Drugs (DMARDs) (e.g., hydroxychloroquine, methotrexate, azathioprine, and mycophenolate mofetil) or new biotechnological therapies [e.g., monoclonal antibodies targeting Tumor Necrosis Factor-alpha (TNF-alpha)] (58). In addition, over the last 5-10 years, rituximab (RTX) has proven to be an effective and relatively safe second-line therapy for both autoimmune and non-malignant lymphoproliferative manifestations (59). Abatacept (a fusion protein of the extracellular domain of CTLA-4 and human IgG1, which prevents antigen-presenting cells from delivering the co-stimulatory signal) has also been used to treat CVID associated autoimmune cytopenia with promising results (60, 61).

In conclusion, autoimmune manifestations, especially cytopenia, and inflammatory diseases are much more frequent in patients with PIDs than in the general population. For this reason, patients with autoimmune anemia, thrombocytopenia, or both should always be screened for PIDs, including CVID. Early recognition and tailored treatments of autoimmune conditions are pivotal to ensure a better quality of life and the reduction of complications associated with CVID. Indeed, thanks to the recent molecular and genetic findings in this immunodeficiency, more targeted approaches are available through precision medicine therapy. For this reason, physicians should raise their awareness about the autoimmune manifestation of PIDs to avoid diagnostic delays and ensure adequate pharmacological therapies.

## Data Availability Statement

The raw data supporting the conclusions of this article will be made available by the authors, without undue reservation.

## Ethics Statement

This study was performed in accordance with the principles of the Helsinki Declaration. Written informed consent was obtained from every subject involved in this study. An informed written consent was obtained for the use of human images (**Figure 4**).

## Author Contributions

IM, FG, AdP, GS, and FR participated in planning the study, drafting the article, critical revision of the article for important intellectual content, and final approval of the article. AP, MW, and CR participated in drafting the article, analysis, and interpretation of data. All authors contributed to the article and approved the submitted version.

## Conflict of Interest

The authors declare that the research was conducted in the absence of any commercial or financial relationships that could be construed as a potential conflict of interest.

## Abbreviations

AIHA, Autoimmune hemolytic anemia; BAFF-R, B cell-activating factor receptor; CD, Cluster of differentiation; CID, Combined immunodeficiency; CVID, Common variable immunodeficiency; CTLA-4, Cytotoxic T-lymphocyte antigen 4; DMARDs, Disease-modifying anti-rheumatic drugs; ESID, European Society for Immunodeficiency; GLILD, Granulomatous and Lymphocytic Interstitial Lung Disease; ICOS, Inducible T Cell Costimulator; ICOS-L, Inducible T Cell Costimulator Ligand; IgRT, Immunoglobulin replacement therapy; Ig, immunoglobulin; ITP, Immune thrombocytopenia; IVIG, Intravenous immunoglobulin; LRBA, Lipopolysaccharide-responsive beige-like anchor protein; PID, Primary immunodeficiency; SEM, Standard error of the mean; pDCs, Plasmacytoid dendritic cell; RTX, Rituximab; TACI, Transmembrane activator and the calcium-modulator and cyclophilin ligand interactor; TLR, Toll-like receptor, TNF-alpha, Tumor Necrosis Factor-alpha; Treg, T regulatory cells.
